# Placenta-derived multipotent mesenchymal stromal cells: a promising potential cell-based therapy for canine inflammatory brain disease

**DOI:** 10.1186/s13287-020-01799-0

**Published:** 2020-07-22

**Authors:** Rogério Martins Amorim, Kaitlin C. Clark, Naomi J. Walker, Priyadarsini Kumar, Kyle Herout, Dori L. Borjesson, Aijun Wang

**Affiliations:** 1 Veterinary Institute for Regenerative Cures and Department of Pathology, Microbiology and Immunology, School of Veterinary Medicine, University of California, Davis, Davis, CA USA; 2 Department of Veterinary Clinics, São Paulo State University “Julio de Mesquita Filho” – UNESP, Botucatu, SP Brazil; 3 Surgical Bioengineering Laboratory, Department of Surgery, School of Medicine, University of California, Davis, 4625 2nd Ave., Research II, Suite 3005, Sacramento, CA 95817 USA; 4 Institute for Pediatric Regenerative Medicine (IPRM), Shriners Hospitals Pediatric Research Center, Northern California, Sacramento, CA USA; 5 Department of Biomedical Engineering, University of California, Davis, CA USA

**Keywords:** Multipotent progenitor cell, Mesenchymal stromal cell, Adipose tissue, Placenta, Canine, Animal model, Immunomodulation, Inflammatory brain disease, Multiple sclerosis, Translational research

## Abstract

**Background:**

Canine inflammatory brain disease (IBD) is a severe inflammatory disorder characterized by infiltration of activated immune cell subsets into the brain and spinal cord. Multipotent mesenchymal stromal cells (MSCs) are a promising therapy for IBD, based on their potent pro-angiogenic, neuroprotective, and immunomodulatory properties. The aims of this study were to compare the immunomodulatory attributes of canine adipose-derived MSCs (ASCs) and placenta-derived MSCs (PMSCs) in vitro. These data will serve as potency information to help inform the optimal MSC cell source to treat naturally occurring canine IBD.

**Methods:**

Indoleamine 2,3 dioxygenase (IDO) activity and prostaglandin E_2_ (PGE_2_) concentration at baseline and after stimulation with interferon gamma (IFNγ) and/or tumor necrosis factor alpha (TNFα) were measured from canine ASC and PMSC cultures. Leukocyte suppression assays (LSAs) were performed to compare the ability of ASCs and PMSCs to inhibit activated peripheral blood mononuclear cell (PBMC) proliferation. IDO activity and PGE_2_; interleukin (IL)-2, IL-6, and IL-8; TNFα; and vascular endothelial growth factor (VEGF) concentrations were also measured from co-culture supernatants. Cell cycle analysis was performed to determine how ASCs and PMSCs altered lymphocyte proliferation.

**Results:**

Activated canine MSCs from both tissue sources secreted high concentrations of IDO and PGE_2_, after direct stimulation with IFNγ and TNFα, or indirect stimulation by activated PBMCs. Both ASCs and PMSCs inhibited activated PBMC proliferation in LSA assays; however, PMSCs inhibited PBMC proliferation significantly more than ASCs. Blocking PGE_2_ and IDO in LSA assays determined that PGE_2_ is important only for ASC inhibition of PBMC proliferation. Activated ASCs increased IL-6 and VEGF secretion and decreased TNFα secretion, while activated PMSCs increased IL-6, IL-8, and VEGF secretion. ASCs inhibited lymphocyte proliferation via cell cycle arrest in the G0/G1 and PMSCs inhibited lymphocyte proliferation via induction of lymphocyte apoptosis.

**Conclusion:**

Our results demonstrate that ASCs and PMSCs have substantial in vitro potential as a cell-based therapy for IBD; however, PMSCs more potently inhibited lymphocyte proliferation by inducing apoptosis of activated lymphocytes. These data suggest that the mechanism by which ASCs and PMSCs downregulate PBMC proliferation differs. Additional studies may elucidate additional mechanisms by which canine MSCs modulate neuroinflammatory responses.

## Background

Over the past decade, stem cell therapy has become a cornerstone in regenerative medicine therapies for many diseases. However, brain and spinal cord diseases represent a challenge in stem cell-based therapy, due to the multiplicity of cell types in the adult central nervous system (CNS) and the precision of cell interactions, in both space and time, required to enhance neuroregeneration [1]. Stem cell therapies for CNS injury are based on cell replacement, via the transplantation of neural progenitor cells, the stimulation of endogenous CNS stem cells, or on improvement of the microenvironment mediated by anti-inflammatory/immunomodulatory paracrine cell effects [2, 3]. However, challenges arise due to a lack of standardization of therapeutic interventions, variability in animal models of disease, alterations in timing and modality of cell application, and a lack of understanding of disease pathology [4].

Multipotent mesenchymal stromal cells (also known as mesenchymal stem cells; MSCs) derived from the bone marrow, adipose tissue, and birth-associated tissues, including umbilical cord, blood cord, amniotic fluid, and placenta, are the most common cell type investigated in cell-based therapy. Several reports have demonstrated positive effects of MSC therapy in a large number of disorders, including brain and spinal cord injuries, in laboratory animals, dogs, and humans [5, 6]. Dogs are increasingly recognized as important animal models for translational medicine because they have naturally occurring brain and spinal cord injuries, such as canine inflammatory brain disease (IBD), similar to multiple sclerosis (MS) in human beings [7–9].

Studies involving MSCs are increasing due to their immunomodulatory, anti-inflammatory, and tissue regenerative properties including the secretion of numerous bioactive molecules leading to tissue regeneration [10, 11]. However, the mechanisms by which MSCs elicit positive effects on the damaged nervous system are not fully characterized. Mechanisms that may play an important role in neuroregeneration include the secretion of growth factors, antiapoptotic factors, neurotrophic factors, cytokines, and extracellular matrix proteins. The immunomodulatory and anti-inflammatory properties of MSCs are also implicated in their ability to protect and repair neurons. Together, these factors promote endogenous neuronal growth, promote neuro/gliogenesis, encourage synaptic connection from damaged neurons, recruit local oligodendrocyte precursors, reduce demyelination, stimulate angiogenesis, decrease apoptosis, reduce oxidative stress, modulate microglial activation, and regulate inflammation by suppressing pathological T, B, and natural killer (NK) cell responses [12–16]. Moreover, they can accelerate a shift from a predominance of pro-inflammatory Th1 cells toward an increase in the anti-inflammatory Th2 cells [17, 18]. Although controversial, some studies have suggested that MSCs can also migrate into the CNS lesion and differentiate into neurons or astrocytes [19, 20].

Canine adult and fetal/neonatal MSCs have been characterized by immunophenotyping and multipotency assays in many studies [21–27]. Fetal/neonatal MSCs, including placenta-derived stem cells, preserve some features of the primitive embryonic layers. They have the potential to differentiate in many tissues [28], have greater proliferative and immunomodulatory capacity and lesser immunogenicity than adult MSCs [28–30], and are neuroprotective [31, 32]. These stem cells can also be easily harvested and expanded due to the availability of a large amount of tissue which is usually discarded at birth. There is no ethical conflict regarding placenta harvest making these stem cell sources attractive targets for banking and regenerative therapies [28, 30]. Nonetheless, a deeper understanding of their immunomodulatory function is still lacking.

**Fig. 1:**
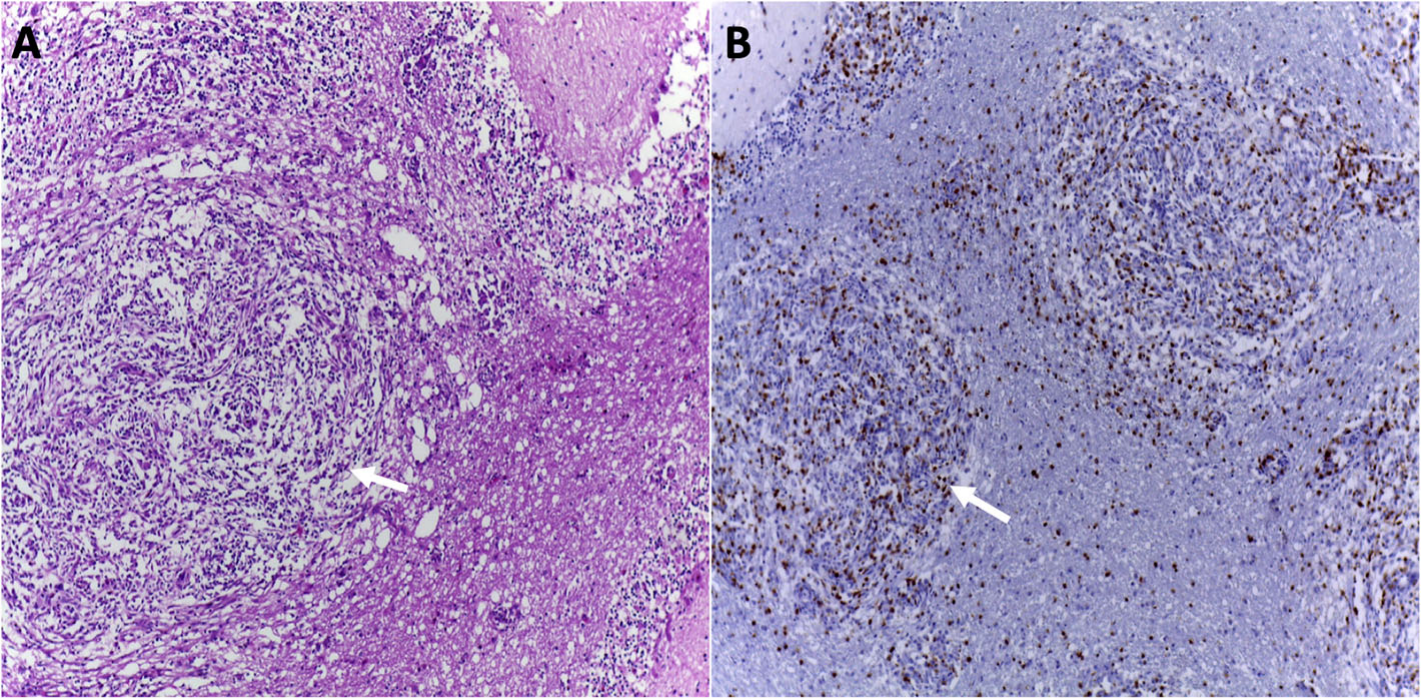
Canine granulomatous meningoencephalitis (GME) in a 2-year-old, female, Miniature Pinscher. **a** Arrow indicates area of granulomatous inflammation (hematoxylin-eosin; × 10). **b** CD3-positive cells in the granulomatous inflammation are indicated (arrow) (immunohistochemistry, DAB, Harris hematoxylin counter stain; × 10)

IBD is a general term used to describe an idiopathic disorder that can be subdivided based on histopathology findings, i.e., granulomatous meningoencephalomyelitis (GME), necrotizing meningoencephalitis (NME), and necrotizing leukoencephalitis (NLE) [33, 34]. Recently, the term meningoencephalomyelitis of unknown origin (MUO) has been proposed to encompass all of these diseases. MUO is presumed to be an autoimmune disease with a genetic predisposition [33]. Immunohistochemical phenotyping of the involved inflammatory cells demonstrates a pivotal role of MHC II+ cells, T cells, and macrophages in GME and NLE [35–37]. GME can be identified by CD3+ lymphocyte infiltration into sites of granulomatous inflammation as shown in a representative clinical case shown in Fig. 1. The pattern of mRNA and protein expression of cytokines and chemokine receptors may be disease specific, such as interferon gamma (IFNγ) and CXCR3 in NME, and IL-17 and CCR2 in GME [34]. Cytokines have also been implicated in the phenotype switching of microglia/macrophages from a classically activated pro-inflammatory type (M1) or into an alternatively activated anti-inflammatory phenotype (M2) in canine inflammatory CNS disease [37]. Thus, manipulating this inflammatory phenotype by cell-based therapy might represent a promising therapeutic approach for MUO [37].

Our goal was to compare the in vitro immunomodulatory ability of 2 types of canine MSCs focusing on secretion profiles and neuroreparative assays relevant for canine IBD. This study evaluated the in vitro potential of canine adipose tissue-derived multipotent mesenchymal stromal cells (ASCs) and placenta-derived multipotent mesenchymal stromal cells (PMSCs) to secrete anti-inflammatory cytokines and to inhibit lymphocyte proliferation, as well as the mechanisms involved in the canine MSC immunomodulatory process.

## Methods

### Canine MSC collection, isolation and culture

#### Adipose tissue-derived mesenchymal stromal cells (ASCs)

Canine low passage (P2-P5) ASCs from 5 dogs were obtained from falciform fat collected from UCD William R. Pritchard Veterinary Medical Teaching Hospital healthy patients undergoing routine abdominal surgery. Fat was collected under an approved Institutional Animal Care and Use Committee and the Clinical Trials Review Board protocol at UCD (protocol number 19605). Fat was processed and canine ASCs were isolated, expanded, cryopreserved, and phenotyped exactly as previously described [38, 39]. Briefly, adipose tissue was minced and digested at 37 °C for 1–2 h using 0.1% collagenase type I and 1% bovine-serum albumin (Worthington, Lakewood, NJ). Centrifugation was performed to remove the remaining lipid layer. The cell pellet was washed several times before being plated in standard medium, Dulbecco’s modified Eagle’s medium (DMEM, Gibco, Invitrogen, Carlsbad, CA) supplemented with 10% FBS (HyClone, Logan, UT), 100 U/mL penicillin and 100 μg/mL streptomycin (ThermoFisher Scientific, Gibco, Pittsburgh, PA).

#### Placenta-derived mesenchymal stromal cells (PMSCs)

Canine low passage (P2-P5) placenta-derived MSCs from 5 dogs were obtained from Dr. Aijun Wang’s laboratory cell bank, University of California Davis Medical Center. Isolation, culture, and full phenotyping of these PMSC lines from discarded term canine placentas was previously described [40]. Briefly, at term, canine placentas were processed by manual dissection and treated with 0.25% trypsin (ThermoFisher) solution for 30 min at 37 °C. Cell pellets were washed and incubated with collagenase IA (1 mg/mL; Sigma-Aldrich, St. Louis, MO) for 45 min at 37 °C. The cell pellet was resuspended and plated onto tissue culture flasks in standard medium.

### Stimulation of canine MSCs with IFNγ and tumor necrosis factor alpha (TNFα)

Cryopreserved MSCs were thawed and culture expanded as previously described [38, 40]. When cells were approximately 70% confluent, they were trypsinized and resuspended in standard medium supplemented with L-tryptophan (Sigma-Aldrich) to a final concentration of 600 μM. These MSCs were seeded at 2 × 10^5^ per well of 24-well plate, with 0.75 mL total media volume per well for the stimulation assays. At plating, MSC was stimulated with IFNγ (50 ng/mL; canine recombinant IFNγ, Kingfisher, St. Paul, MN) or TNFα (50 ng/mL; canine recombinant TNFα, Kingfisher) or a dual stimulation with both IFNγ (50 ng/mL, Kingfisher) and TNFα (50 ng/mL, Kingfisher). Stimulated MSCs were cultured for 4 days at which time supernatants were collected, frozen, and stored at − 80 °C for mediator quantification. Stimulation protocols using IFNγ and TNFα were optimized by using a dose titration strategy and collection at multiple timepoints for mediator quantification [data not shown]. The optimized strategy is consistent with previous reports on IFNγ and TNFα secretion by activated canine MSCs [38, 39].

### Canine leukocyte suppression assay (LSA)

Canine leukocyte suppression assay (LSAs) were performed exactly as previously described [38]. In brief, peripheral blood was collected from healthy dogs 55 pounds or larger between 1 and 8 years old, into tubes containing sodium heparin (Vacutainer®, BD Biosciences) via jugular venipuncture. PBMCs were isolated using a discontinuous Ficoll gradient and were plated with irradiated (10 Gy, Varian 2100C linear accelerator, Varian Medical Systems, Inc., Palo Alto, CA) allogeneic canine ASCs or PMSCs in standard medium (DMEM with 10% FBS, 1% penicillin/streptomycin, supplemented with 600 μM L-tryptophan) [38]. PBMCs were activated with 5 μg/mL concanavalin A (ConA; Sigma-Aldrich). PBMCs and irradiated MSCs were co-cocultured at a ratio of 5:1 in direct contact.

To determine the role of contact, cells were plated in transwell dishes (Corning 0.4 μM polycarbonate membrane 24-well plate; Corning, NY, USA) with MSCs plated in the plate bottom and PBMCs in the insert. To determine the role of IDO and PGE_2_ in MSC-mediated inhibition of lymphocyte proliferation, inhibitory agents were used in LSA co-cultures. Indomethacin, a cyclooxygenase (COX) inhibitor, was used to chemically block PGE_2_ production (Cayman Chemical Co., Ann Arbor, MI, 1μM). Alternatively, 1-methyl-DL-tryptophan (1-MT), an IDO competitive inhibitor, was used to partially inhibit IDO activity. Indomethacin was added to LSA assays during plating at a concentration of 10 μM (Sigma-Aldrich) as previously described [38] to determine the role of PGE_2_ on MSC-mediated immunosuppression. Additionally, at plating, 1-MT (Sigma-Aldrich) was added at a concentration of 1 mM as previously described [41] to determine the role of IDO activity on MSC mediated immunosuppression.

After 3 days of co-culture, wells were treated with 1 mM Bromodeoxyuridine (BrdU, BD Biosciences). Twenty-four hours post BrdU treatment, leukocytes were collected, and cells were stained with a viability dye (Fixable Viability Dye Fluor**®** 780; eBioscience, San Diego, CA) and anti-canine CD3 conjugated to Alexa Fluor**®** 488 (clone CA17.2A12; Leukocyte Antigen Biology Lab, UCD). Leukocytes were stained for nuclear BrdU incorporation (APC BrdU Flow Kit, BD Biosciences) per manufacturer directions and analyzed by flow cytometry (Cytomics FC500). Fold reduction of leukocyte proliferation by MSCs was normalized to stimulated donor PMBCs for any given experiment. For cell cycle analysis, 7-aminoactinomycin D was added to cultures per manufacturer’s instructions (APC BrdU Flow Kit; BD Biosciences) and analyzed by flow cytometry on days 1–4. Flow cytometry data were analyzed using FlowJo flow cytometry software (Tree Star, Inc.). Data was normalized and presented as a reduction of each respective PBMC donor.

At the time of leukocyte collection, culture supernatant was collected, centrifuged, and stored at − 80 °C for the measurement of secreted mediators.

### Quantification of mediator secretion

Frozen aliquots of supernatants collected from MSCs stimulated with IFNγ/TNFα and from LSA co-cultures using two technical replicates from each assay were used to quantify PGE_2_ and IDO activity. Canine PGE_2_ was quantified using an ELISA kit per manufacturer directions (Prostaglandin E2 Express EIA kit (Monoclonal); Cayman Chemical Company, Ann Arbor, MI) [38]. To assess IDO activity, a biochemical assay was performed on frozen supernatants as previously described [42] to quantify the conversion of tryptophan to *N*-formyl kynurenine mediated by IDO. In brief, culture media was treated with 30% trichloroacetic acid (Sigma), and Ehrlich’s reagent (1% *p-*dimethylaminobenzaldehyde in glacial acetic acid, Sigma) was mixed and read at 490 nm on a microplate reader (Synergy HT Multi-Mode Gen5 software) [38].

Supernatants from LSAs co-cultures were used to measure concentrations of interleukin (IL)-2, IL-6, and IL-8; vascular endothelial growth factor (VEGF); and TNFα. IL-2, IL-6, and IL-8 and were quantified via Quantibody^VR^ Canine Cytokine Array (RayBiotech cat# QAC-CYT-1). Negative controls were prepared from wells containing only media. The array was performed according to the manufacturer’s instructions, and the resulting glass slide was scanned using a GenePix 4000B microarray scanner (Molecular Devices). Collected images were quantified using GenePix^VR^ Pro 6 acquisition and analysis software, and further plotting of standard curves and analysis was performed using Microsoft Excel. TNFα was measured from supernatants using ELISA kits (Canine TNFα DuoSet, R&D Systems) per manufactures instructions. All ELISA samples were read on a Synergy HT Multi-Mode microplate reader with Gen5 software (Biotek, Winooski, VT, USA).

### Statistical analysis

Results are presented as mean and standard error. Data were tested for normality using the Shapiro-Wilk normality test (GraphPad InStat version 3.06 for Windows, La Jolla, CA). Data were analyzed using non-parametric Mann-Whitney-Wilcoxon *t* test (GraphPad InStat version 3.06 for Windows, La Jolla, CA). *p* < 0.05 was considered statistically significant.

## Results

### Canine MSCs increase IDO and PGE_2_ secretion in response to IFNγ and TNFα stimulation

**Fig. 2:**
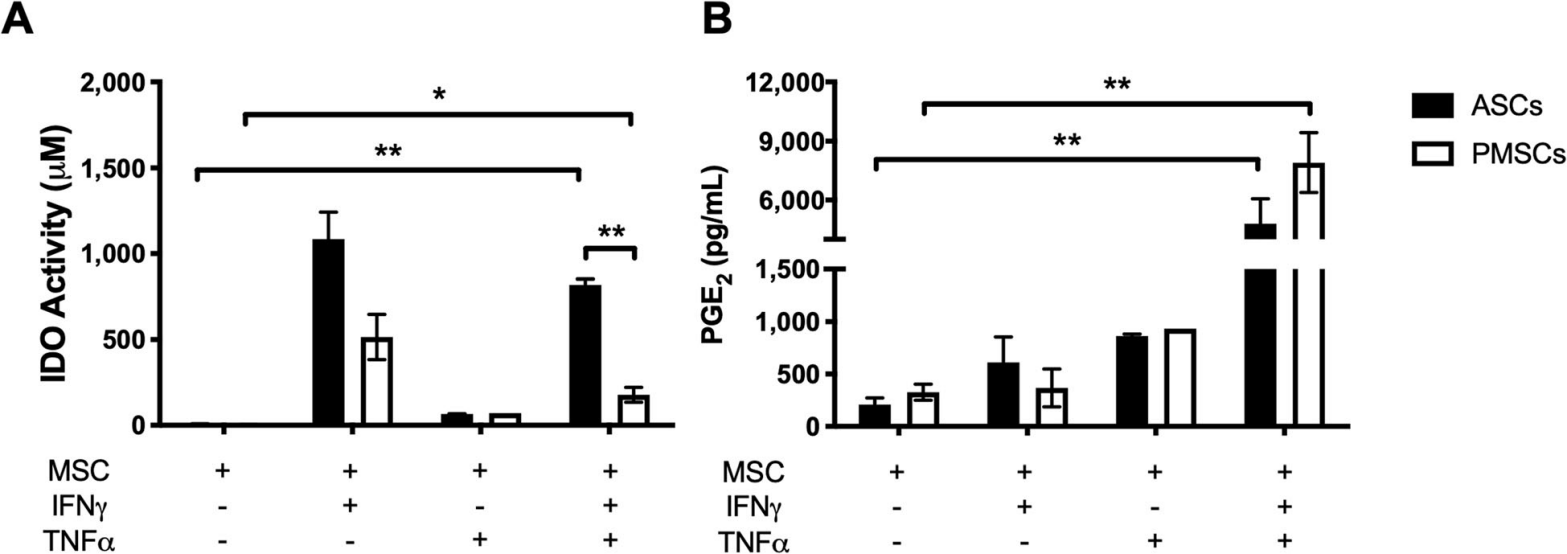
Direct stimulation of canine ASCs and PMSCs leads to production of IDO and PGE_2_. Canine adipose-derived MSCs (*ASCs*) and placenta-derived MSCs (*PMSCs*) secrete increased levels of indoleamine 2,3 dioxygenase (IDO) activity and prostaglandin E_2_ (PGE_2_) in response to direct stimulation using recombinant interferon gamma (IFNγ) and tumor necrosis factor alpha (TNFα*)*. **a** IDO activity is directly proportional to the conversion of tryptophan to *N*-formyl kynurenine. ASCs and PMSCs increase IDO activity in response to dual stimulation with IFNγ and TNFα. IFNγ is the main contributor to the production of IDO. IFNγ- and TNFα-stimulated ASCs promote significantly higher levels of IDO activity as compared to PMSCs. **b** Canine ASCs and PMSCs produce comparable levels of prostaglandin E_2_ (PGE_2_) in response to dual stimulation with IFNγ and TNFα. TNFα is the major contributor to MSC-mediated PGE_2_ production. Data presented as mean and standard error. *p < 0.05, **p < 0.01, ***p < 0.001. IDO, indoleamine 2,3 dioxygenase; IFNγ, interferon gamma; MSC, mesenchymal stem cell; PGE_2_, prostaglandin E_2_; TNFα, tumor necrosis factor alpha

MSC immunomodulatory functions occur in part through the secretion of bioactive factors. MSCs were activated through direct stimulation to determine the immunomodulatory potential using recombinant pro-inflammatory mediators known to be relevant in IBD. Stimulation with IFNγ alone increased IDO activity, while stimulation with TNFα alone predominantly stimulated PGE_2_ production by both ASCs and PMSCs (Fig. 2a, b). The use of both stimulation agents resulted in a synergistic effect IDO activity and PGE_2_ production; therefore, dual stimulation using both IFNγ and TNFα was performed in canine ASC and PMSC cultures. Dual stimulation with both IFNγ and TNFα resulted in increased PGE_2_ production and IDO activity (Fig. 2a, b). Canine ASCs, however, secreted significantly more IDO than PMSCs after 4 days of stimulation with canine recombinant IFNγ and TNFα (Fig. 2a; *p* = 0.0079). Dual stimulation of canine ASCs and PMSCs resulted in comparable increases in PGE_2_ secretion (Fig. 2b). IDO secretion was exclusively dependent on IFNγ with no synergistic increase after the addition of TNFα; however, PGE_2_ secretion was augmented with dual stimulation.

### Canine ASCs and PMSCs inhibit activated PBMC proliferation through distinct mechanisms in a contact-dependent manner

**Fig. 3:**
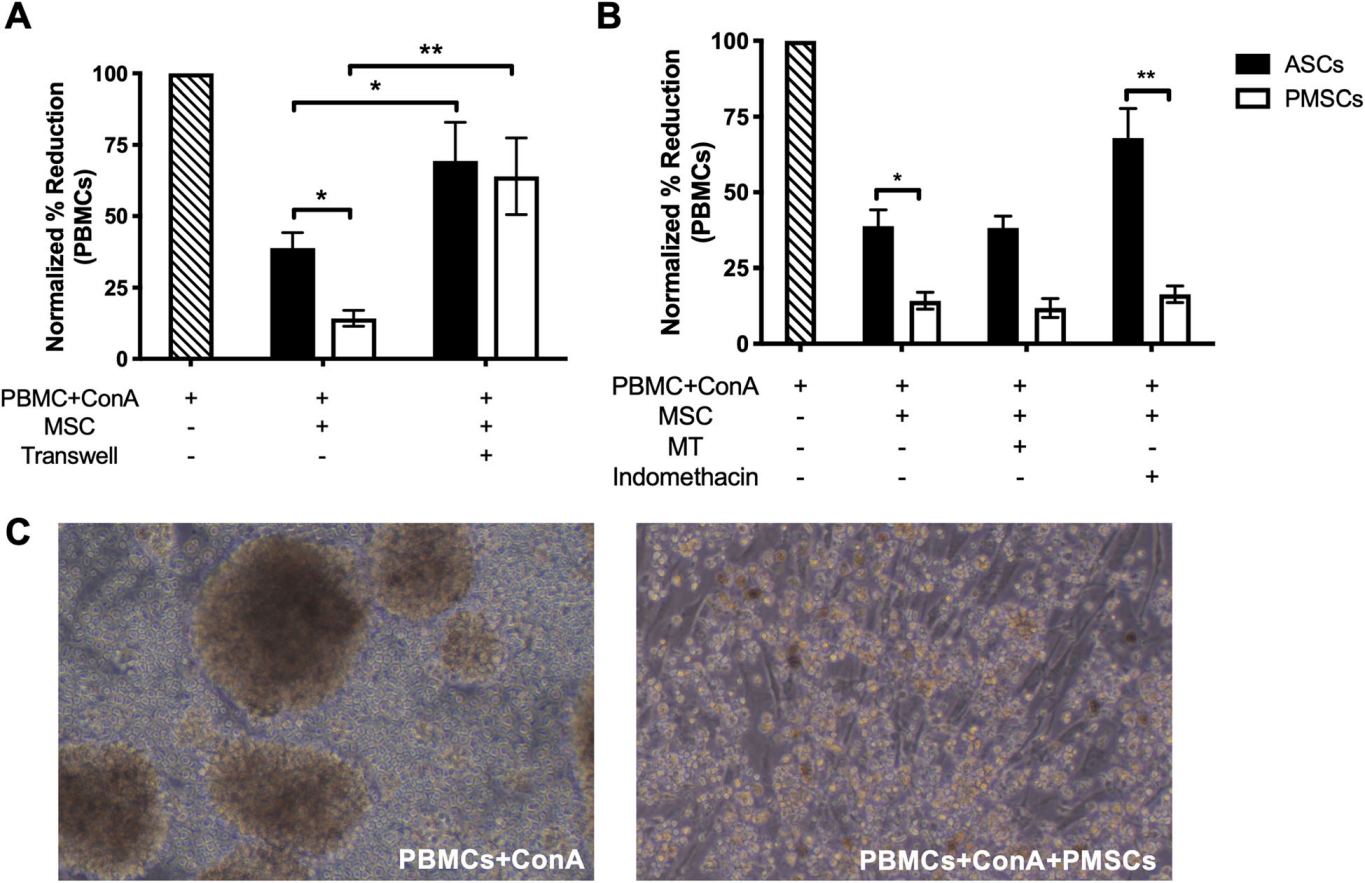
Canine ASCs and PMSCs inhibit lymphocyte proliferation in a contact-dependent manner. Canine adipose-derived MSCs (*ASCs*) and placenta-derived MSCs (*PMSCs*) possess immune-suppressive functions. **a** Canine ASCs and PMSCs co-incubated with stimulated peripheral blood mononuclear cells (*PBMCs*) suppress lymphocyte proliferation. PMSCs significantly decrease lymphocyte proliferation more potently as compared to ASCs. Transwells were added to remove physical contact between MSCs and mitogen (ConA) stimulated PBMCs. Lymphocyte proliferation increased when ASCs and PMSCs were not in direct contact with activated PBMCs. **b** To determine the role of indoleamine 2,3 dioxygenase (*IDO*) and prostaglandin E_2_ (*PGE*_*2*_) in MSC-mediated immunosuppression 1-methyl-DL-tryptophan (1-MT) and indomethacin was added to co-cultures to block each respective mediator. Blocking of IDO activity using 1-MT resulted in no alterations in MSC-mediated suppression of PBMC proliferation. Blocking PGE_2_ using indomethacin resulted in a loss of lymphocyte inhibition by ASCs but no effects were observed by PMSCs. PBMC stimulation is normalized and data is presented as a reduction to each respective donor. Representative photomicrographs of ConA stimulated PBMCs and PMSC LSA conditions (**c**). Data presented as mean and standard error. *p < 0.05, **p < 0.01, ***p < 0.001. 1-MT, 1-methyl-DL-tryptophan; ConA, concanavalin A; IDO, indoleamine 2,3 dioxygenase; LSA, leukocyte suppression assay; MSC, mesenchymal stem cell; PBMCs, peripheral blood mononuclear cells; PGE_2_, prostaglandin E_2_

We have previously reported that canine ASCs reduce mitogen-activated PBMC proliferation in LSA co-cultures [38]. Both canine ASCs and PMSCs in direct contact with activated PBMCs inhibited lymphocyte proliferation; however, PMSCs more potently inhibited lymphocyte proliferation compared to ASCs (Fig. 3a, *p* = 0.0127). LSA co-cultures performed within a transwell to remove direct MSC-PBMC cell contact resulted in marked restoration of PBMC proliferation regardless of MSC tissue source (Fig. 3a). These data suggest that canine MSCs reduce activated lymphocyte proliferation in part via direct cell-cell contact.

MSC mediated immunomodulation in dogs also occurs through the secretion of bioactive factors [38, 39, 43, 44]. PGE_2_ and IDO have been implicated as crucial mechanisms by which MSCs downregulate inflammatory responses. To evaluate the role of PGE_2_, the COX inhibitor indomethacin was used to block PGE_2_ synthesis and secretion. The competitive inhibitor 1-MT was used to block the functional properties of IDO. Blocking PGE_2_ led to a significant reduction of ASC-mediated inhibition as compared to PMSCs (Fig. 3b, *p* = 0.0043). Blocking IDO, however, lead to no alterations in ASC- or PMSC-mediated suppression of PBMC proliferation (Fig. 3b). Representative photomicrographs of stimulated PBMCs and a PMSC LSA are shown in Fig. 3c. No morphological changes were noted between canine ASC and PMSC LSA conditions.

**Fig. 4:**
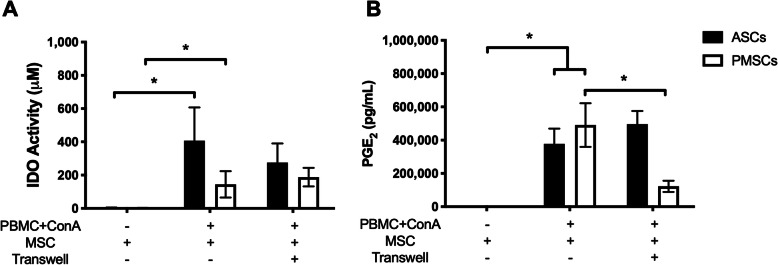
Indirect stimulation of canine ASCs and PMSCs in leukocyte suppression assay (LSAs) leads to production of IDO and PGE_2_. Canine adipose-derived MSCs (*ASCs*) and placenta-derived MSCs (*PMSCs*) produce similar secretory mediators when co-cultured in contact with activated peripheral blood mononuclear cells (PBMCs). **a** ASCs and PMSCs increase indoleamine 2,3 dioxygenase (*IDO*) activity in the presence of mitogen (ConA) activated PBMCs. Removal of direct cellular contact did not alter IDO activity by either ASCs or PMSCs. **b** Production of prostaglandin E_2_ (*PGE*_*2*_) occurred in both standard and transwell conditions. Canine PMSCs secrete significantly greater levels of PGE_2_; however, when direct contact was removed using transwells, only canine PMSCs drastically reduced PGE_2_ production. No observable changes occur when contact was removed in ASC co-cultures. Data presented as mean and standard error. *p < 0.05, **p < 0.01, ***p < 0.001. ConA, concanavalin A; IDO, indoleamine 2,3 dioxygenase; MSC, mesenchymal stem cell; PBMCs, peripheral blood mononuclear cells; PGE_2_, prostaglandin E_2_

Secretion of PGE_2_ and IDO in LSA supernatants was determined in standard LSA conditions (MSC-PBMC contact) and in transwell conditions (no cell contact). ASCs and PMSCs secreted comparable levels of IDO when co-cultured in direct contact with mitogen-activated PBMCs (Fig. 4a). Removing direct MSC-PBMC cell contact had no effect on IDO secretion from either MSC source (Fig. 4a). Both ASCs and PMSCs also secreted comparable high levels of PGE_2_ when co-cultured with stimulated PBMCs as compared to basal secretions; however, when direct PBMC cell contact was removed, only PMSCs secretion of PGE_2_ significantly decreased (Fig. 4b).

### Activated canine PMSC and ASC secretion profiles

**Fig. 5:**
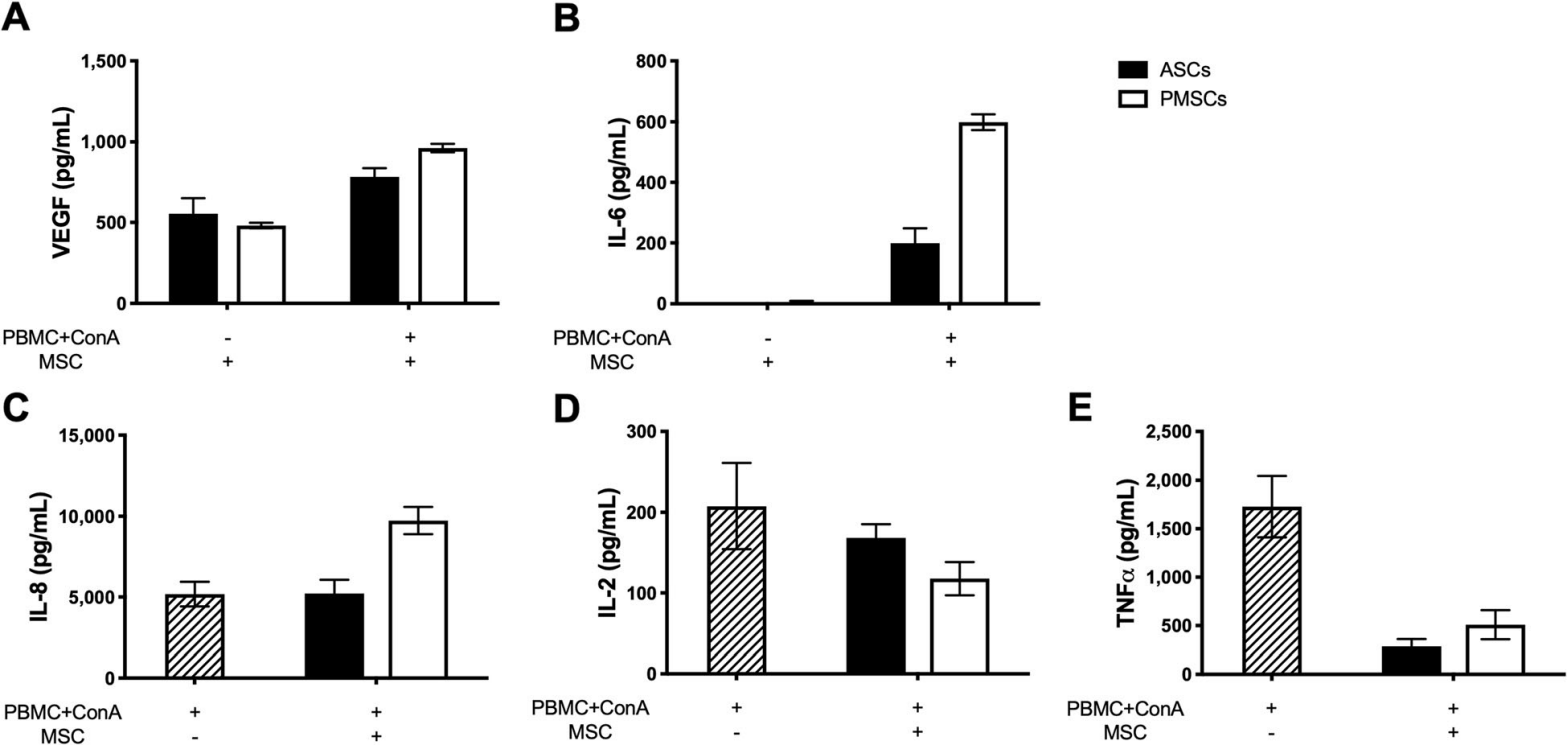
Bioactive factors associated with canine ASC and PMSC mediated immunosuppression differs. Canine adipose-derived MSCs (*ASCs*) and placenta-derived MSCs (*PMSCs*) increase production of vascular endothelial growth factor (VEGF) and IL-6. **a** Both canine ASCs and PMSCs mildly increased production of VEGF when co-cultured with mitogen (ConA) activated peripheral blood mononuclear cells (PBMCs). **b** Canine ASCs and PMSCs upregulated IL-6 production; however, PMSCs secreted higher levels of IL-6 as compared to ASCs. **c** Production of IL-8 by canine ASCs was comparable to basal levels of IL-8 produced by stimulated PBMCs; however, PMSCs increased levels of IL-8 greater than basal levels. Additionally, regulation of inflammatory mediators by canine ASCs and PMSCs was similar. **d** IL-2 production from stimulated PBMCs was mildly reduced by both canine ASCs and PMSCs. **e** Canine ASCs and PMSCs inhibit production of tumor necrosis factor alpha (*TNFα*) by stimulated PBMCs to comparable levels. Data presented as mean and standard error. ConA, concanavalin A IL, interleukin; TNFα, tumor necrosis factor alpha; VEGF, vascular endothelial growth factor

Protein concentrations were measured to determine the mediators present in LSA supernatant after co-incubation of activated PBMCs with PMSCs and ASCs. Mediators implicated in the immunomodulatory functions of MSCs were measured and compared to basal levels from unstimulated MSCs. Statistical significance was not achieved; however, trends were observed suggesting that activated ASCs and PMSCs secrete VEGF (Fig. 5a) and IL-6 (Fig. 5b). Additionally, the mediator IL-8, which is secreted by both MSCs and PBMCs, was measured. Unstimulated MSCs did not secrete detectable levels of IL-8 (data not shown). Basal levels of IL-8 from activated PBMCs are comparable to concentrations when ASCs were added to co-culture; however, PMSCs produced greater levels of IL-8 (Fig. 5c). MSCs have also been shown to suppress inflammatory responses through the reduction of pro-inflammatory cytokines. Both ASCs and PMSCs displayed a trend of reduced IL-2 concentrations (Fig. 5d), and both tissues sources reduced TNFα to similar concentrations (Fig. 5e).

### ASCs inhibit lymphocyte proliferation through cycle arrest, while PMSCs induce apoptosis

**Fig. 6:**
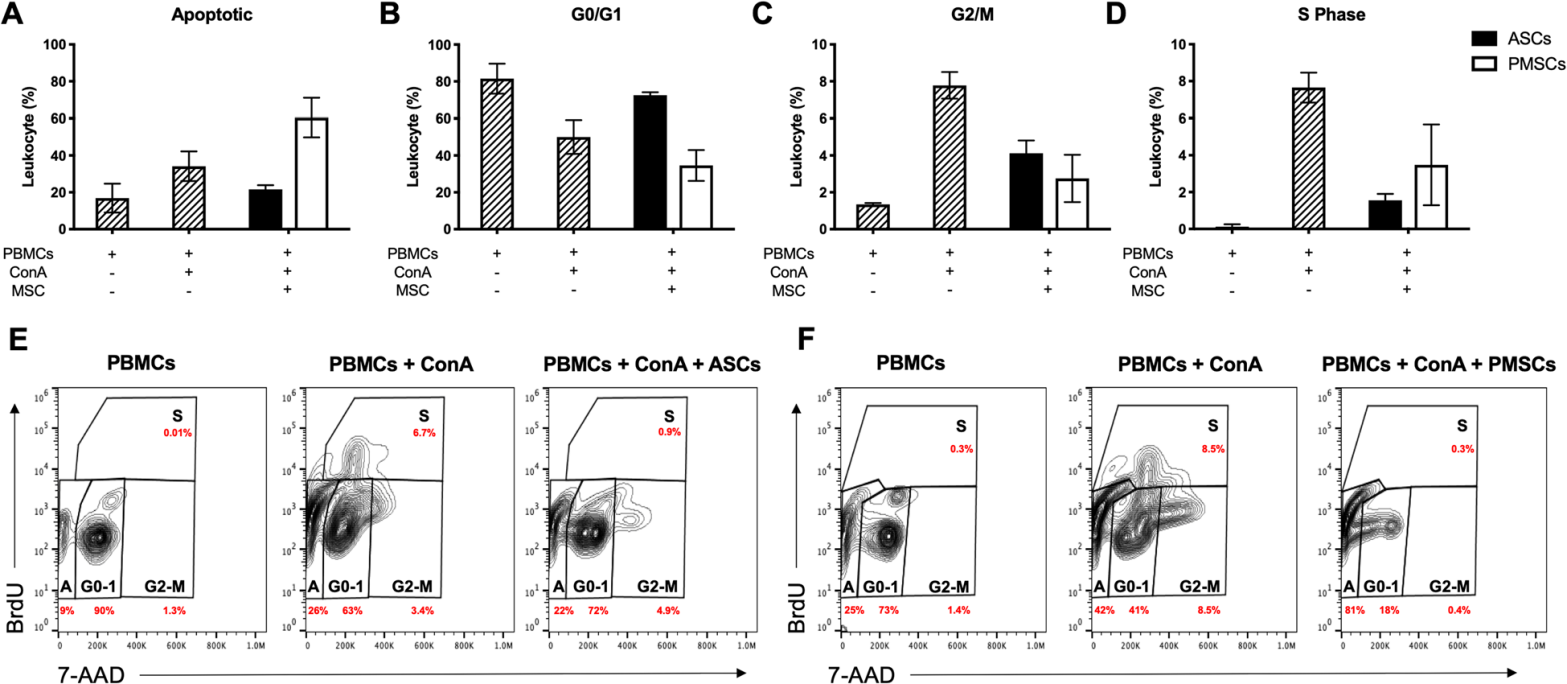
Inhibition of lymphocyte proliferation by canine ASCs and PMSCs occurs through different mechanisms. Cell cycle analysis was performed using peripheral blood mononuclear cells (PBMCs) and *BrdU* 5-bromo-2′-deoxyuridine and 7-aminoactinomycin D was measured. Unstimulated PBMCs and mitogen (ConA) activated PBMCs were used as controls. **a** Canine PMSCs inhibit lymphocyte proliferation by inducing apoptosis. Alternatively, canine ASCs caused cell cycle arrest which is demonstrated by PBMCs accumulating in G_0_/G_1_ (**b**) and hindering cells from entering G2/M (**c**) or DNA synthesis (S phase) (**d**). Representative images of cell cycle flow scatter plots and gating strategies for leukocyte DNA content (7-AAD) and proliferation via BrdU incorporation of PBMC controls (**e**, **f**) and co-incubations with canine ASCs (**e**) and PMSCs (**f**) are shown. *BrdU*, 5-bromo-2′-deoxyuridine and 7-aminoactinomycin D; ConA, concanavalin A; LSA, leukocyte suppression assay; MSC, mesenchymal stem cell

The inhibition of lymphocyte proliferation can be secondary to cell cycle arrest or the induction of apoptosis. Our findings suggest canine MSCs varied in how they inhibited lymphocyte proliferation, depending on the tissue source (adipose or placenta). Our data indicates canine PMSCs induced lymphocyte apoptosis (Fig. 6a) while canine ASCs result in induced lymphocyte arrest in the G0/G1 phase of cell cycle (Fig. 6b). Both tissue sources result in decreased lymphocyte entry into G2/M (Fig. 6c) and S phase (Fig. 6d) of the cell cycle; however, each decreases entry through alternative mechanisms. Representative images of cell cycle flow scatter for unstimulated PBMCs (Fig. 6e, f), ConA stimulated PBMCs (Fig. 6e, f) and LSA conditions for canine ASCs (Fig. 6e) and PMSCs (Fig. 6f) are shown.

## Discussion

Companion animals are increasingly being utilized as naturally occurring large animal disease models to evaluate the use stem cell-based therapies. Veterinary species suffer from many diseases that closely resemble the pathophysiology of human diseases, making them valuable translational models for preclinical data. The dog has been used to evaluate MSC therapy for the treatment of several inflammatory conditions including osteoarthritis, spinal cord injury, inflammatory bowel disease, and graft-versus-host disease [19, 45–47].

Murine experimental autoimmune encephalomyelitis (EAE) is the most commonly used animal model to study MS. However, EAE does not reproduce all clinical, pathological, or immunological features of human disease [48]. Canine MUO may be useful as a naturally occurring model of MS, given neuroimmunological similarities of these diseases, including the upregulation of IFNγ, IL-17, and MHC-II expression in the nervous system [5, 7, 34, 49–51]. Moreover, the genetic association of MHC-II found in dogs with MUO is present in MS [7]. MS is suggested to be mediated by Th1 and Th17 lymphocytes, leading to demyelination and axonal injury [52, 53]. Although the demyelination noted in MS is not present in NME, fulminant or non-prototypic acute variants of MS, such as Marburg variant, Balo’s concentric sclerosis, and acute disseminated encephalomyelitis, closely resemble the pathological features of canine NME [7]. The focal and widespread forms of GME, consistent with a delayed hypersensitivity reaction, are also consistent with MS [5]. Cytokine expression in brain lesions of NME and GME display increased levels of interferon gamma (IFNγ) in NME and IL-4 and IL-17 in GME [34]. IL-17 and IFNγ production by T lymphocytes is also associated with active disease in MS patients [53]. In addition, CSF in dogs with MUO showed increased levels of CCL19 chemokine, also expressed in neuroinflammatory diseases such as MS / EAE, suggesting similar neuroimmunological events [54].

MSCs are an attractive target for neurodegenerative disease therapies due to their potent neuroprotective, regenerative, and immunomodulatory properties. While most clinical studies utilize adult-derived sources of MSCs, the placenta is a unique source of MSCs that maintain unique functional properties for therapeutic use as compared to adult tissue-derived MSCs. We found that MSCs from adult and fetal tissue sources modulate PBMC proliferation through multiple mechanisms. Both canine ASCs and PMSCs inhibit activated lymphocyte proliferation through a primarily cell-cell contact-mediated mechanism. Notably, PMSCs more potently inhibited lymphocyte proliferation as compared to ASCs in vitro. Inhibition of PBMC proliferation by PMSCs occurred through the induction of apoptosis, while ASCs induced cell cycle arrest. Canine ASCs and PMSCs secreted high levels of both PGE_2_ and IDO through direct stimulation with IFNγ and TNFα or through indirect stimulation in our mixed co-culture assay. For PMSCs, when cell-cell contact was removed, a restoration of lymphocyte proliferation and a decrease of PGE_2_ secretion were observed. Interestingly, blocking PGE_2_ production in PMSC cultures using a COX-inhibitor did not restore lymphocyte proliferation. These findings suggest that MSC-PBMC cellular contact is primarily responsible for the production of PGE_2_ by PMSCs; however, production of this mediator is not directly responsible for induction of lymphocyte apoptosis. Additionally, PMSCs produce IDO but regardless of removing cellular contact or blocking IDO via competitive inhibitor, no effect on PBMC proliferation was observed. Collectively, this data highlights the importance of cellular contact for PMSC-mediated immunosuppression and initiation of PBMC apoptosis.

Similarly, in ASCs, when direct cellular contact was removed with PBMCs, lymphocyte proliferation was restored; however, this contact was not needed for PGE_2_ secretion. Inhibiting PGE_2_ production with indomethacin led to increased lymphocyte proliferation by ASCs. These data suggest that for canine ASCs, there are two mechanisms leading to PBMC cell cycle arrest, one that is contact-dependent and one that is PGE_2_-dependent. As observed in PMSCs, inhibiting IDO with 1-MT showed no effect on ASC mediated immunosuppression. Removing MSC-PBMC contact did not alter IDO activity in canine ASC co-cultures. These findings suggest that IDO does not play a significant role in canine MSC-mediated immunosuppression from either tissue source.

PGE_2_ has been shown to play a key role in MSC-mediated immunosuppression in humans, cats, dogs, and horses [18, 55]. PGE_2_ inhibits production of IFNγ and IL-2 and induces T regulatory cells [56]. Decidual stem cells also suppress alloreactivity through induction of T regulatory cells in a contact-dependent manner; however, involvement of programmed cell death 1 (PD-1), IDO, PGE_2_, and IFNγ still plays a role in this suppression [57]. These data suggest that our findings in canine fetal PMSCs are comparable to human studies. Interestingly, we observed no alterations in immunosuppression by the secretion of IDO, which has been reported in both human adult and fetal-derived tissue sources. Here we report canine MSCs produce IDO when activated; however, this does not seem to be a critical mediator in lymphocyte suppression.

Though not statistically significant, our data suggest that both ASCs and PMSCs secrete VEGF and IL-6. Additionally, PMSCs, trended toward secreting higher levels of IL-6 and IL-8 as compared to ASCs. There is also a trend toward a reduction of IL-2 production by both ASCs and PMSCs. Both tissue sources reduced the production of the pro-inflammatory mediator TNFα. Canine ASC mediators closely recapitulate studies in human ASCs, suggesting that the dog will serve as a useful translational model to evaluate therapeutic applications of MSCs [58]. The ability of canine MSCs from either tissue source to secrete immunomodulatory mediators and suppress inflammatory cytokines indicates these cells are attractive targets for canine IBD therapeutics.

MSC-immune cell contact, namely in the presence of IFNγ and TNFα, has been shown to upregulate PDL-1, vascular cell adhesion molecule 1 (VCAM-1), and inflammatory cytokine-induced adhesion molecule 1 (ICAM-1) and augments the secretion of soluble mediators [59–61]. Additionally, ICAM-1 by feline ASCs has been shown to play a critical role in ASC-T cell adhesion and mediates T cell proliferation [61]. Contact-dependent inhibition of immune cell function has been suggested to play a more important role in local immunosuppression [57]. It has also been shown that blockade of ICAM-1 and VCAM-1 ablates MSC-mediated immunosuppression which highlights the potential mechanistic role of adhesion molecules by MSCs [60]. The role of ICAM-1/LFA ligand has been shown to play a critical role in feline ASC-mediated immunosuppression through induction of G0-G1 cell cycle arrest [61]. Feline ASCs also induce cell cycle arrest by utilizing PGE_2_ which is comparable to our findings in dog MSCs, suggesting ICAM-1 may also play a significant role in canine MSC mediated immunosuppression. Therapies targeting adhesion molecules have been used for numerous diseases including MS, using a drug that targets α_4_β_1_-integrin [62]. Our work has demonstrated the critical role of MSC-immune cell contact in immunoregulation; however, the exact mechanism by which these interactions occur will need to be addressed in future studies. Additionally, a mechanistic comparison of canine and human PMSCs will need to be performed to fully establish the utility of this model.

Taken together, our data suggest that canine ASCs and PMSCs possess immunoregulatory properties. However, the mechanisms by which this immunoregulation occurs differs. Though both cell sources are immunosuppressive, PMSCs displayed more potent ability to decrease activated T cell proliferation. Additionally, PMSCs induce PBMC apoptosis while ASCs induce cell cycle arrest. This highlights the need to consider disease pathology when selecting MSC tissue sources for selected therapies. More studies are needed to understand the mechanistic differences between cell source and immune cell interactions. From our studies, we suggest that PMSCs may be a novel therapeutic source for neurodevelopmental and neurodegenerative diseases.

## Conclusions

The findings from this study demonstrate that canine ASCs and PMSCs have robust immunoregulatory potential. The mechanism of immune suppression by each cell source differs, in that PMSCs induce apoptosis of activated lymphocytes and ASCs induce cell cycle arrest. Secretome profiles of activated MSCs from each source also differed; however, PMSCs notably more potently inhibit lymphocyte proliferation. While each tissue source holds great potential as a cell-based therapy for IBD, PMSCs may be an ideal tissue source for many neurodegenerative diseases in both animals and humans. Additional studies will be needed to further elucidate the mechanism by which canine MSCs modulate neuroinflammatory responses.

## Data Availability

The datasets used and/or analyzed during the current study are available from the corresponding author on reasonable request.

## Abbreviations

1-MT: 1-Methyl-DL-tryptophan
ASC: Adipose-derived mesenchymal stromal cell
BrdU: 5-Bromo-2'-deoxyuridine
ConA: Concanavalin A
CNS: Central nervous system
EAE: Experimental autoimmune encephalomyelitis
FBS: Fetal bovine serum
GME: Granulomatous meningoencephalomyelitis
IBD: Inflammatory brain disease
ICAM-1: Inflammatory cytokine-induced adhesion molecule 1
IDO: Indoleamine 2,3 dioxygenase
IFNγ: Interferon gamma
IL: Interleukin
LSA: Leukocyte suppression assay
MS: Multiple sclerosis
MSC: Multipotent mesenchymal stromal cells
MUO: Meningoencephalomyelitis of unknown origin
NK: Natural killer cells
NLE: Necrotizing leukoencephalitis
NME: Necrotizing meningoencephalitis
PBMC: Peripheral blood mononuclear cell
PBS: Phosphate buffered saline
PD-1: Programmed cell death 1
PGE_2_: Prostaglandin E2
PMSC: Placenta-derived mesenchymal stromal cell
TNFα: Tumor necrosis factor alpha
VCAM-1: Vascular cell adhesion molecule 1
VEGF: Vascular endothelial growth factor
